# Benthic macrofauna bioturbation and early colonization in newly flooded coastal habitats

**DOI:** 10.1371/journal.pone.0196097

**Published:** 2018-04-25

**Authors:** Thomas Valdemarsen, Cintia O. Quintana, Sandra W. Thorsen, Erik Kristensen

**Affiliations:** Department of Biology, University of Southern Denmark, Odense M, Denmark; University of Sydney, AUSTRALIA

## Abstract

How will coastal soils in areas newly flooded with seawater function as habitat for benthic marine organisms? This research question is highly relevant as global sea level rise and coastal realignment will cause flooding of soils and form new marine habitats. In this study, we tested experimentally the capacity of common marine polychaetes, *Marenzelleria viridis*, *Nereis (Hediste) diversicolor* and *Scoloplos armiger* to colonize and modify the biogeochemistry of the newly established Gyldensteen Coastal Lagoon, Denmark. All tested polychaetes survived relatively well (28–89%) and stimulated carbon dioxide release (TCO_2_) by 97–105% when transferred to newly flooded soils, suggesting that soil characteristics are modified rapidly by colonizing fauna. A field survey showed that the pioneering benthic community inside the lagoon was structurally different from the marine area outside the lagoon, and *M*. *viridis* and *S*. *armiger* were not among the early colonizers. These were instead *N*. *diversicolor* and *Polydora cornuta* with an abundance of 1603 and 540 ind m^-2^, respectively. Considering the species-specific effects of *N*. *diversicolor* on TCO_2_ release and its average abundance in the lagoon, we estimate that organic carbon degradation was increased by 219% in the first year of flooding. We therefore conclude that early colonizing polychaetes modify the soils and may play an important role in the ecological and successional developments, e.g. C cycling and biodiversity, in newly flooded coastal ecosystems. Newly flooded soils have thus a strong potential to develop into well-functioning marine ecosystems.

## Introduction

Future sea levels are predicted to rise 2–16 mm yr^-1^ as a consequence of climate change, implying 0.2–1.2 m higher average sea levels by year 2100 [1]. Inevitably, some low-lying coastal areas will be permanently flooded in the future [2]. These include coastal soils with low elevation and gentle slopes towards the inland and areas that are currently below mean sea level and kept dry by drainage and dikes. Engineering techniques such as coastal realignment [3] may be implemented to protect valuable assets in coastal zones, which will also result in the formation of newly flooded habitats. Only few studies have, until now, illustrated how such newly flooded coastal areas will develop as habitat for marine organisms [4,5]. This research question is nevertheless highly important for the future management of coastal zones. For instance, it is critical to know if flooded areas will remain barren for an extended time, or if they will rapidly develop into productive ecosystems with high ecological and recreational value. Knowing the fate of coastal areas after flooding may provide decision makers with sufficient knowledge for either implementing appropriate mitigation measures, or allowing nature to run its course.

Benthic macrofauna such as polychaetes and bivalves are the foundation for food webs and ecological functioning in coastal marine ecosystems [6–8]. Various organisms, including fish, crustaceans and birds, depend on benthic macrofauna as a food source [9,10]. The rate of development of benthic macrofaunal communities in newly flooded areas can, therefore, be expected to exert a critical control on total biodiversity [4,9,10]. Furthermore, marine benthic macrofauna have been shown to modulate sediment composition and biogeochemical processes through bioturbation activities [11]. Bioturbation is well known to influence ecosystem functionality [12,13] by altering redox-driven microbial pathways [14,15] with consequences for organic matter degradation [16,17], and exchange of O_2_, CO_2_ and nutrients between sediment and overlying water [7,18,19]. Thus, it is likely that pioneering benthic macrofauna may modulate overall ecological developments in newly flooded coastal soils, with significant impacts on primary and secondary productivity as well as succession trajectories.

Previous studies imply various levels of benthic macrofauna colonization and succession in constructed marine wetlands depending on larval dispersion strategy, species eco-physiology and local environmental conditions. The most critical environmental factors controlling the colonization and succession of benthic fauna are distance to water exchange (i.e. water circulation) and characteristics of the substratum [4,20]. On constructed mudflats devoid of vegetation and open to lateral migration of juveniles and adults, initial colonization may occur within days and macrofauna communities may reach stable composition within months [21,22]. In restored saltmarshes, benthic macrofauna colonization can also be rapid, with benthic fauna communities corresponding to ambient conditions within a time scale of months to a few years [23,24], but in other cases it may take several years to decades [4,25]. For instance, Garbutt et al. [20] noted that benthic macrofauna colonization in a 21 ha salt marsh created on flooded agricultural soil occurred rapidly in newly accreted sediments, while parts of the saltmarsh without sediment accretion and with agricultural remains were azoic for an extended time. Accordingly, there is no consensus on how initial colonization of benthic macrofauna develops to a stable community structure and how species behave in different types of flooded terrestrial soils.

In this study, we combine experimental results and field observations to assess the capacity of various macrofauna species to colonize and modify newly flooded coastal habitats. In laboratory experiments, we tested how selected species of polychaetes with different behavioral traits survive and perform basic functions (e.g. bioirrigation) in terrestrial soils shortly after flooding with seawater. In the field, we followed the early succession of benthic fauna within the first year after flooding of the Gyldensteen Coastal Lagoon on Northern Fyn, Denmark and compared the results to communities in the adjacent marine area. The behavior of experimental species was then related to their success or lack hereof during the first year of macrofauna colonization in two sites in the newly flooded lagoon. The species-specific impact of successful polychaete pioneers on soil chemistry was assessed, and formed the basis for an evaluation of how these species can promote further succession of macrobenthic communities and ecological functioning in newly flooded habitats.

## Materials and methods

### Study area

**Fig 1:**
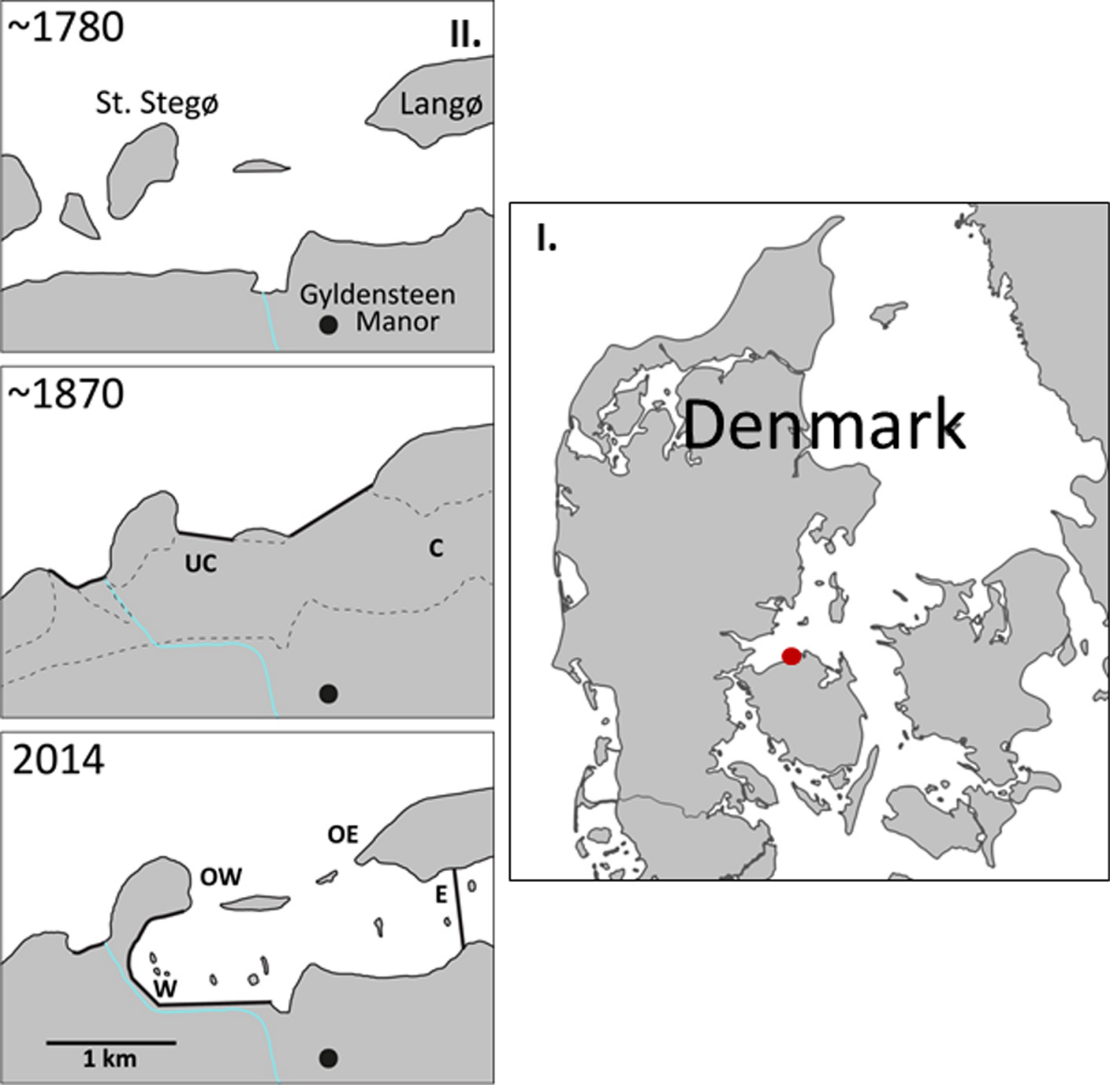
Map of the study area. I: Map of Denmark with location of study area (red circle: 55.574°N, 10.140°E) indicated. II: Gyldensteen Coastal Lagoon area in its original state (upper) redrawn from a 1780 map; just after land reclamation in 1871 (middle) with locations of the 2014 soil sampling sites: uncultivated (UC) and cultivated (C); and the area after flooding in 2014 (lower) with indications of macrofaunal sampling stations: outside west (OW), west (W), outside east (OE) and east (E) stations.

The Aage V. Jensen Nature Foundation purchased 616 ha of land at Gyldensteen Strand on northern Fyn, Denmark in 2011 (Fig 1). Most of the land had been reclaimed around 1870 and used for agricultural purposes for more than 140 years [26]. A shallow marine lagoon with a total surface area of 214 ha was restored as a managed coastal realignment project, where new dikes were constructed along the inland perimeter and flooding occurred through three northern openings in the original dikes (Fig 1). Before flooding, the area was primarily composed of cultivated soils, but with small uncultivated patches near the western seaward dikes. The uncultivated soil had never been used for agriculture due to its low elevation and lack of drainage. Wild grasses and herbs were allowed to grow and a dense root layer penetrated down to 5 cm depth. The soil below the root layer was densely packed clay. The cultivated soil was in 2013 largely used for grass seed production with perennial ryegrass (*Lolium perenne*). Analysis of soil cores collected at several stations in the cultivated area revealed that agricultural activities, i.e. ploughing, were evident down to 0.5–1 m depth [27]. The soil in this layer had a homogenous texture. The Gyldensteen Coastal Lagoon was flooded on 29 March, 2014. The resulting coastal lagoon is shallow (mean depth about 0.5 m) and microtidal (about ± 20 cm compared to mean sea level), but larger water level fluctuations (up to ± 100 cm) may occur during exceptional wind events. The salinity in the lagoon follows the salinity in the ambient marine environment in Kattegat (20–30) and there are no freshwater inputs to the system, except for a few minor drainage channels in the southern part.

### Laboratory experiment to evaluate fauna colonization potential

Soil cores were sampled from two stations inside the Gyldensteen Strand area in March 2014, four weeks before the area was flooded (Fig 1). One station was located in uncultivated soil (UC) and the other in cultivated soil (C). The large difference in the structure of the two soil types was evident from the higher porosity (0.82–0.97) and carbon content (16%) in UC compared to C (porosity: 0.53–0.58 and carbon content: 1%) in the upper 2 cm [27]. Fifteen soil cores were sampled randomly to a depth of 20 cm at each station using 30 cm long and 8 cm internal diameter transparent acrylic core liners and closed in both ends with rubber stoppers. Vegetation protruding more than 0.5–1 cm from the surface in UC cores was cut-off right after sampling. Seawater for the experiment (salinity of 29) was collected in the marine area adjacent to Gyldensteen Strand (Fig 1).

Soil cores were randomly divided in the laboratory into four groups, one for each species treatment and another for defaunated controls, which were placed into four separate 70 L tanks filled with seawater. Each group consisted of three UC and three C cores. The water reservoir in each tank was vigorously aerated by air pumps and about 20% was renewed with fresh seawater every week during the experiment. The flooded soil cores were left to acclimatize for 28 days in darkness at constant temperature (15°C) to facilitate seawater percolation into the soil. Hereafter a light source was mounted over the soil cores (12/12 hours light/darkness, ca. 600 µE m^-2^ s^-1^ at the soil surface) to stimulate growth of benthic microalgae as a potential food source for polychaetes during the experiment.

Three species of polychaetes with different functional traits, *Marenzelleria viridis*, *Nereis (Hediste) diversicolor* and *Scoloplos armiger*, were used in the experiment. *M*. *viridis* is an invasive species in Europe, which has spread via pelagic larvae [28,29]. It is a head-up deposit feeder inhabiting 10–30 cm deep J-shaped blind-ended burrows [17,30]. *N*. *diversicolor* is a known pioneer species after disturbance or restoration of shallow marine habitats through dispersal of benthic larvae and juveniles [24,31,32]. It is a surface deposit or suspension-feeder inhabiting 10 cm deep and usually U-shaped burrows with two openings [33]. *S*. *armiger* is a cosmopolitan species found in widely different habitats ranging from shallow brackish estuaries to the deep sea [34]. It is a head-down deposit feeder inhabiting 5–15 cm deep I-shaped blind ended burrows [35,36]. The three species were chosen because they are early colonizers and common in the shallow marine areas outside Gyldensteen Coastal Lagoon. A pilot study conducted in April 2013 with 6 stations along the coast showed average abundances ± SE (n = 4) of 772 ± 168, 552 ± 220 and 48 ± 30 ind m^-2^ for *M*. *viridis*, *N*. *diversicolor* and *S*. *armiger*, respectively.

Intact and healthy polychaetes were collected 31 days after flooding of soil cores and weighed before they were added to the cores the following day. Each of the three polychaetes species were added to three core replicates for each of the two soil types. Time of polychaete addition is referred to as t = 0. *M*. *viridis* and *N*. *diversicolor* were added in the same abundance (6 ind per core = 1194 m^-2^), and due to its smaller size, the abundance of *S*. *armiger* was twice as high (12 ind per core = 2387 m^-2^). Accordingly, we aimed for similar polychaete biomass in all treatments (171–217 g m^-2^; Table 1).

**Table 1:** Water content, density, porosity and loss on ignition (LOI) in control cores from uncultivated (UC) and cultivated (C) soils.

	UC				C			
Depth (cm)	Water content (%)	Density (g cm^-3^)	Porosity	LOI (%)	Water content (%)	Density (g cm^-3^)	Porosity	LOI (%)
0–1	72 ± 4	0.32 ± 0.06	0.83 ± 0.01	24.1 ± 2.7	33 ± 2	1.14 ± 0.07	0.57 ± 0.01	3.8 ± 0.3
1–2	65 ± 0	0.42 ± 0.01	0.79 ± 0.01	23.9 ± 1.2	25 ± 1	1.31 ± 0.03	0.44 ±0.02	3.1 ± 0.1
2–3	59 ± 2	0.53 ± 0.03	0.75 ± 0.01	n.a.	24 ± 1	1.43 ±0.01	0.44 ±0.02	n.a.
3–4	53 ± 2	0.61 ± 0.03	0.70 ± 0.03	21.1 ± 1.8	22 ± 1	1.45 ± 0.03	0.40 ± 0.00	2.9 ± 0.1
4–5	53 ± 3	0.64 ± 0.06	0.70 ± 0.03	n.a.	21 ± 0	1.50 ± 0.01	0.40 ± 0.01	n.a.
5–6	41 ± 2	0.86 ± 0.04	0.59 ± 0.03	n.a.	20 ± 0	1.51 ± 0.02	0.37 ± 0.01	n.a.
6–8	38 ± 1	0.90 ± 0.05	0.54 ± 0.01	10.3 ± 0.1	20 ± 0	1.50 ± 0.00	0.37 ± 0.01	2.9 ± 0.0
8–10	33 ± 1	0.93 ± 0.03	0.46 ± 0.01	n.a.	19 ± 1	1.45 ±0.04	0.34 ± 0.02	n.a.
10–12	31± 2	1.01 ±0.06	0.46 ± 0.03	n.a.	19 ± 1	1.47 ± 0.01	0.35 ± 0.02	n.a.
12–14	33 ± 1	1.00 ± 0.01	0.49 ± 0.02	7.6 ± 0.5	19 ± 1	1.39 ±0.01	0.33 ± 0.01	2.8 ± 0.1
14–16	38 ± 2	0.84 ± 0.01	0.51 ± 0.03	n.a.	20 ± 0	1.52 ± 0.02	0.37 ± 0.00	n.a.
16–18	36 ± 2	0.91 ± 0.03	0.52 ± 0.04	7.0 ± 0.3	21 ± 1	1.43 ± 0.04	0.39 ± 0.01	2.9 ± 0.1

Values are given as average ± standard error (n = 3). n.a.: not analyzed.

Soil O_2_ uptake (SOU), release of TCO_2_ = H_2_CO_3_ + HCO_3_^-^ + CO_3_^2-^), and exchange of dissolved inorganic N (DIN = NH_4_^+^ + NO_3_^-^ + NO_2_^-^) and P (DIP = PO_4_^3-^) by the soil were measured 4 days before addition of polychaetes and regularly (1–2 times per week) over the following 4 weeks. Initial water samples were taken from the headspace of all cores, before they were sealed and incubated for ca. 4 hours in darkness under constant water circulation. The cores were then opened and final water samples were taken. Samples of 3 mL were preserved with saturated HgCl_2_ (10 µl per mL) and analyzed for TCO_2_ by flow injection analysis [37]. Samples for DIN and DIP were stored frozen (-20°C) until analysis on Flow injection Analyzer. O_2_ change in the headspace was measured with a fiberoptic O_2_ dipping probe. Area specific (core surface area in m^2^) SOU TCO_2_, DIN and DIP fluxes were calculated from concentration changes over time in overlying water during flux incubations (mmol m^-2^ d^-1^).

The bioirrigation activity of the polychaetes was determined by Br^-^ tracer incubations [38]. The water reservoir in every tank was enriched with NaBr to a final concentration of ~8 mM two days before the experiment was terminated. The area-specific bioirrigation volume was calculated from the accumulated porewater Br^-^ inventory corrected for molecular Br^-^ diffusion measured in defaunated control cores [17,39]. The maximum bioirrigation depth (i.e. maximum burrow depth) was determined as the deepest layer where the concentration of Br^-^ was significantly elevated above the background.

The experiment was terminated 27–30 days after polychaete addition. The soil cores were sectioned in 1 cm intervals to 6 cm depth and in 2 cm intervals to 18 cm depth. Every depth layer was carefully searched to recover surviving polychaetes before the soil was used for other analyses. A soil subsample of ~3–4 g from every depth was taken for the determination of soil characteristics. Soil bulk density was determined as the dry weight of a known volume of soil dried at 105°C for 48 h. Water content and porosity were calculated based on the weight loss after drying. Total organic matter was determined in 6 soil depths by loss on ignition (LOI) after combusting dry sediment 5 h at 520°C. Porewater from the remaining portion of wet soil from each depth interval was extracted by centrifuging (10 min, ca. 500g) in double centrifuge tubes equipped with a GF/C-filter. After centrifuging, the soil was sieved through 1 mm mesh to check once more for presence of worms. Porewater was stored frozen (-20°C) until analysis for SO_4_^2-^ and Br^-^ by liquid ion chromatography.

### Initial macrofauna colonization in the Gyldensteen coastal lagoon after flooding

Benthic macrofauna was sampled at two stations (W and E) in opposite ends inside the lagoon and two stations outside the lagoon (OW and OE) (Fig 1). Stations W and E had cultivated soil similar to C soil from the laboratory experiment, since the agriculture practice had been similar throughout cultivated area in the lagoon [27]. A total of 6 samplings were conducted at both stations inside the lagoon during the first year after the flooding, monthly from July to October 2014 and later on February and May 2015. Sampling was more occasional outside the lagoon with station OW visited once before (October 2013) and three times after flooding (April, September 2014 and September 2015). Station OE was only sampled on October 2013 and April 2015. Only a single fauna sampling was done in UC soil (October 2014). Four macrofauna samples (>25 cm deep) were taken during each sampling using a 15 cm diameter stainless steel sampler. The samples were sieved through a 1 mm mesh on site, and retained material was transferred to plastic jars and preserved with 4% buffered formalin. Samples were sorted in the laboratory and all recovered macrofauna was preserved in 70% ethanol. Macrofauna was identified to lowest possible taxonomic level and counted.

### Calculations and statistics

To verify significant differences and interactions between the soil types (UC and C) and species treatment (Defaunated Control, *M*. *viridis*, *N*. *diversicolor* and *S*. *armiger*), two-way ANOVA was performed on solute exchange data from day 0 (after the addition of worms) and onwards. The same test was adopted to verify differences in species recovery, burrow depth and area/weight-specific bioirrigation, but without the control treatment. Normality of the data and homogeneity of variances were checked before applying two-way ANOVA tests. Pair-wise *post-hoc* Tukey tests were done after significant differences were detected by ANOVA. Comparison of early colonizing macrofaunal communities (i.e. abundance and species composition) from the stations inside (W and E) with those outside (OW and OE) were based on non-metrical multidimensional scaling (MDS), where the distance between points was defined by Bray-Curtis similarity ranking on squared root transformed data. One-way analysis of similarity (ANOSIM) was performed to test differences in abundances and species composition between groups formed in MDS analysis, i.e. stations inside and outside the lagoon. Similarity analysis (SIMPER) was applied to identify the species that contributed most to the differences between groups detected in MDS. Vertical profiles of soil density, water content, porosity, SO_4_^2-^ and Br^-^ were not tested statistically, since they were determined for a series of layers in individual replicate cores and therefore were not independent. Relationships between bioirrigation intensity from Br^-^ incubations and stimulation of total benthic metabolism measured as TCO_2_ fluxes and SOU was explored by linear regressions. All statistical tests were performed with a significance level of 0.05 using Sigmaplot 12.5 and Primer 6 package.

## Results

### Soil characteristics

Bulk density was low (0.31–0.42 g cm^3^) in the upper 2 cm of UC soil, followed by a sharp increase with depth reaching up to 1.0 g cm^-3^ below 5 cm (Table 1). Water content, porosity and LOI in UC soil followed the same pattern and were higher in the upper 2 cm (65–72%, 0.79–0.83 and ~24%, respectively), reaching levels of 31–41%, 0.46–0.59 and 7.0–10.0%, respectively below 5 cm depth (Table 1). There was less marked depth variation in the different parameters in C soil (Table 1). From 0 to 18 cm soil depth, bulk density ranged from 1.1–1.5 g cm^-3^, water content from 19–33%, porosity from 0.33–0.57and LOI from 2.8–3.8% (Table 1).

### Polychaete survival and bioirrigation

Polychaete recovery at the end of the experiment was not different between soil types, but varied according to species, with lower recovery for *M*. *viridis* (22–44%) than for *N*. *diversicolor* (55–78%) and *S*. *armiger* (86–89%) (F_2,17_ = 69.0, p <0.001, Table 2). A search by the end of the experiment revealed 15 living individuals of *M*. *viridis*, 3 of *N*. *diversicolor* and 2 of *S*. *armiger* in the bottom of the incubation tanks. Unfortunately, it was impossible to identify whether they escaped from UC or C treatments, since cores of both soil types were incubated in the same tanks. Accordingly, a markedly higher proportion of *M*. *viridis* (42%) than *N*. *diversicolor* and *S*. *armiger* (3–8%) escaped the cores at some point during the experiment, suggesting slightly higher survival of *M*. *viridis* than indicated by the recovery reported in Table 1.

**Table 2:** Number and biomass (wet weight) of *Marenzelleria viridis* (*Mar*), *Nereis diversicolor* (*Ner*) and *Scoloplos armiger* (Sco) added to uncultivated (UC) and cultivated (C) soil cores. Recovery of polychaetes, maximum depth of burrows, area- and weight-specific bioirrigation rates for the three species of polychaetes observed by the end of the experiment.

	UC			C		
	*Mar*	*Ner*	*Sco*	*Mar*	*Ner*	*Sco*
**Added individuals (core**^**-1**^**)**	6	6	12	6	6	12
**Individual biomass (mg ind**^**-1**^**)**	207 ± 23	178 ± 1	72 ± 3	211 ± 14	148 ± 14	74 ± 3
**Total biomass (g m**^**-2**^**)**	217 ± 31	213 ± 16	171 ± 4	204 ± 13	177 ±5	175 ± 5
**Recovery (%)**	28 ± 6^Aa^	56 ± 22^Aa^	89 ± 7^Ab^	44 ± 11^Aa^	79 ±6^Aa^	86 ± 3^Ab^
**Max. burrow depth (cm)**	3.8 ± 0.3*	5.3 ± 0.2*	5.2 ± 0.3*	14.3 ± 1.3*	9.0 ± 1.2*	10.3 ± 1.3*
**Area-specific bioirrigation** **(L m**^**-2**^ **d**^**-1**^**)**	1.5 ± 0.8^Aa^	6.3 ± 1.1^Aa^	5.3 ± 2.0^Aa^	7.4 ± 0.3^Ba^	6.7 ± 0.8^Ba^	9.8 ± 1.1^Ba^
**Weight-specific bioirrigation** **(mL g**^**-1**^ **d**^**-1**^**)**	207 ± 23*	178 ± 1*	72 ± 3*	211 ± 14*	148 ± 14*	74 ± 3*

Values are given as average ± standard error (n = 6–12 for average biomass and n = 3 for remaining statistics). Capital and lower case letters in right panels represent the grouping of data obtained by 2-way ANOVA followed by Tukey *post hoc* analysis. Capital letters indicate significant difference between UC and C. Lower case letters indicate significant difference between treatments (Con, *Mar*, *Ner* and *Sco*).

* indicates significant interaction between the factors soil type and core treatment.

**Fig 2:**
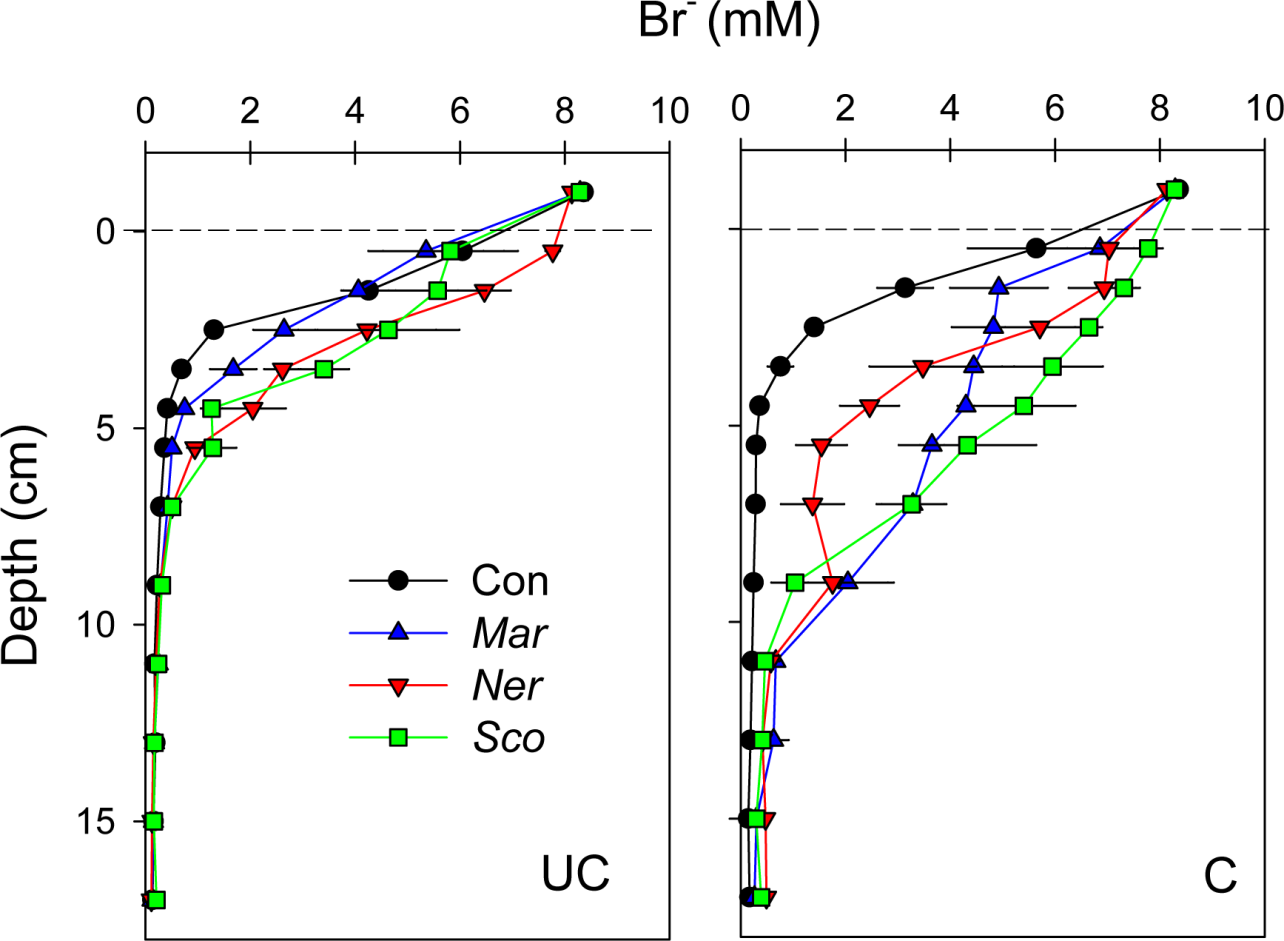
Depth profiles of Br^-^ in uncultivated (UC) and cultivated (C) soils (upper and lower graphs, respectively) without (Con) or with polychaetes added (*Mar*: *Marenzelleria viridis*, *Ner*: *Nereis diversicolor* and *Sco*: *Scoloplos armiger*). Dashed lines indicate the sediment-water interface. Error bars indicate standard error (n = 3).

The Br^-^ tracer experiment showed similar diffusion-controlled profiles in defaunated cores of both soil types, with Br^-^ decreasing rapidly from 5.5–6.0 to 0.7 mM between 0.5 and 3.5 cm depth, and with constant Br^-^ level of about 0.2 mM below (Fig 2). Maximum bioirrigation depth based on Br^-^ profiles varied from 4–5 cm in UC soil to 9–10 cm in C soil for all three species (Table 2). Br^-^ levels in the bioirrigated zone varied from 3 to 8 mM in C soil while concentrations ranged from 1 to 6 mM in UC soils (Fig 2). Accordingly, the area-specific bioirrigation was significantly higher in C compared to UC soil (F_1,17_ = 14.3, p = 0.003), but there was no significant difference between the rates of the three species (F_2,17_ = 3.8, p = 0.052, Table 2).

### Polychaete impact on soil biogeochemistry

**Fig 3:**
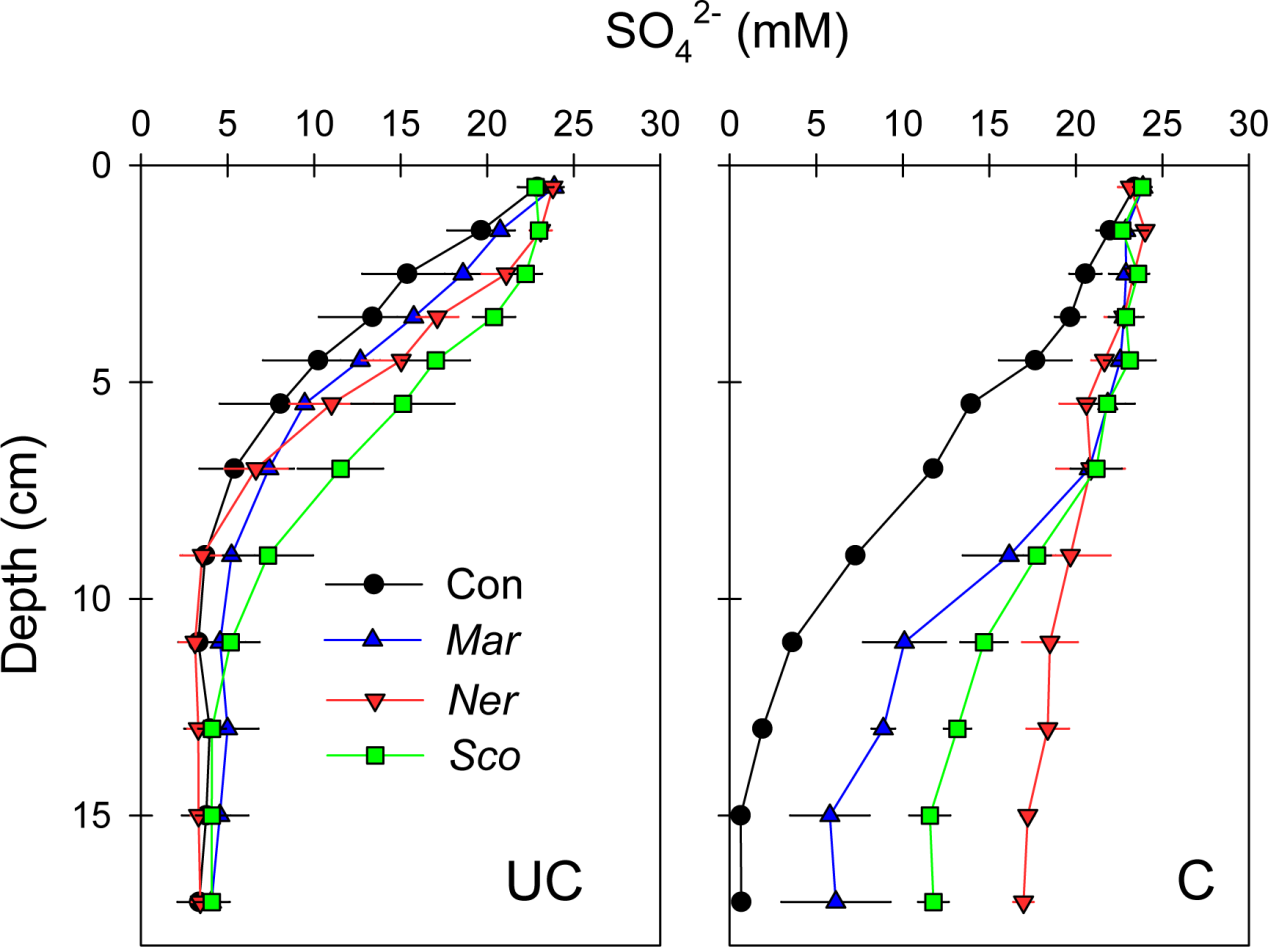
Depth profiles of porewater SO_4_^2-^ in uncultivated soil (UC) and cultivated soil (C) flooded with seawater, without (Con) or with polychaetes added (*Mar*: *Marenzelleria viridis*, *Ner*: *Nereis diversicolor* and *Sco*: *Scoloplos armiger*). Error bars indicate standard error (n = 3).

The vertical distribution of SO_4_^2-^ in porewater was clearly impacted by the presence of polychaetes; particularly in C soil (Fig 3). SO_4_^2-^ decreased in UC control cores, from ~23 mM in surface soil to ~6 mM at 5 cm depth, coinciding with the transition from roots to dense clay, and was constant below. The presence of polychaetes in UC soil elevated SO_4_^2-^ moderately in the upper 7 cm compared to controls; highest for *S*. *armiger* and lowest for *M*. *viridis*. In control cores with C soil, SO_4_^2-^ decreased from ~23 mM at the surface to 5 mM at 12 cm depth. Porewater SO_4_^2-^ in C soil with polychaetes was considerably higher than in controls for all depths, and was almost similar to overlying water down to 7 cm depth and then decreased gradually towards the bottom, with highest levels in *N*. *diversicolor* and lowest in *M*. *viridis* treatments.

**Fig 4:**
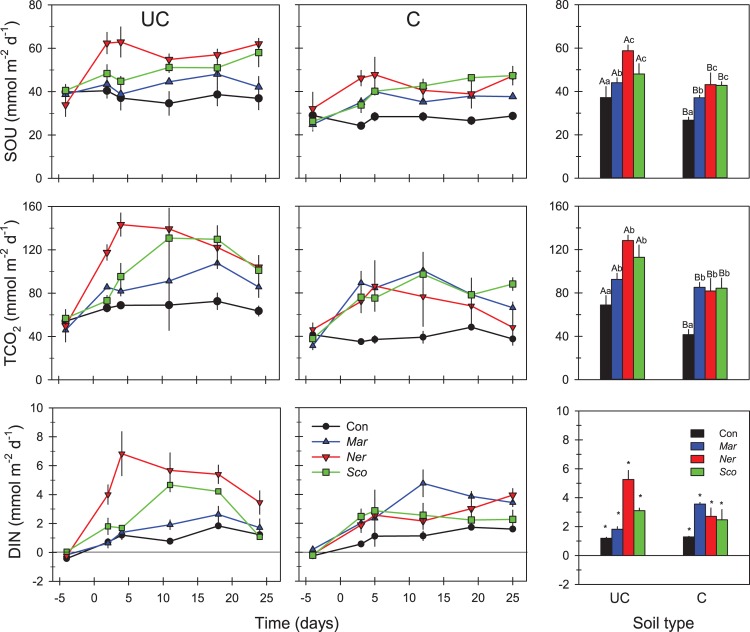
Sediment O_2_ uptake (SOU) and exchange of TCO_2_ and DIN in flooded uncultivated (UC) and cultivated (C) soil without (Con) and with added polychaetes (*Mar*: *Marenzelleria viridis*, *Ner*: *Nereis diversicolor* and *Sco*: *Scoloplos armiger*, respectively). Left and middle panels show temporal patterns and right panels show averages (from time of polychaete addition [t = 0] to end). Error bars indicate SE (n = 3). Capital and lower case letters in right panels represent the grouping of data obtained by 2-way ANOVA followed by Tukey post hoc analysis. Capital letters indicate significant difference between UC and C. Lower case letters indicate significant difference between core treatments (Con, *Mar*, *Ner* and *Sco*). * indicates significant interaction between the factors soil type and core treatment.

The flux incubation performed before the addition of polychaetes confirmed that cores of the same soil type had similar SOU and TCO_2_ and nutrient effluxes (Fig 4). SOU and TCO_2_ fluxes in control cores remained relatively stable over the whole experiment at average levels in UC and C soil of 37 and 27 mmol O_2_ m^-2^ d^-1^, and 69 and 41 mmol CO_2_ m^-2^ d^-1^, respectively (Fig 4). DIN fluxes in control cores showed a slow and gradual increase for both soils, from slightly negative fluxes initially (-0.2 to -0.4 mmol m^-2^ d^-1^) to positive DIN efflux by the end (1.2 to 1.6 mmol m^-2^ d^-1^). No exchange of PO_4_^3-^ between soil cores and overlying water was detected in any treatment. The addition of polychaetes immediately stimulated SOU, TCO_2_ and DIN effluxes (Fig 4). While SOU remained stable throughout the experiment for faunated UC and C soil, TCO_2_ and DIN fluxes decreased toward the end for both soil types. Time averaged SOU and TCO_2_ fluxes from day 0 onwards were significantly higher in UC than in C soil type (F_1,142_ = 52.4 and 26.7, respectively, p <0.001) (Fig 4 and Table 3). Fluxes of TCO_2_ in both soil types were affected similarly by all three polychaete species (63–105%, F_3,142_ = 13.8, p <0.001). However, SOU in both UC and C soils was affected more by *N*. *diversicolor* and *S*. *armiger* (29–61%) than by *M*. *viridis* (F_3,142_ = 25.6, p <0.001) (Fig 4 and Table 3). DIN fluxes had significant interaction (F_3,142_ = 4.3, p = 0.006) between the two tested factors (soil type and core treatment) and therefore *post hoc* comparisons were not applicable. Despite the significant interaction, it is important to mention that DIN fluxes in UC soil were highly stimulated by *N*. *diversicolor* (342%), 2 to 6-fold higher than the DIN enhancement by other polychaetes in UC and C soil (Fig 4 and Table 3).

**Table 3:** Flux increase (%) for treatments with polychaetes added (*Mar*: *Marenzelleria viridis*, *Ner*: *Nereis diversicolor* and *Sco*: *Scoloplos armiger*) in relation to defaunated controls (Con).

	**UC**				**C**			
	Con mmol m^-2^ d^-1^	*Mar* %	*Ner* %	*Sco* %	Con mmol m^-2^ d^-1^	*Mar* %	*Ner* %	*Sco* %
**TCO**_**2**_ **flux**	68.9 ± 8.7	34	86	64	41.4 ± 4.7	105	97	103
**SOU**	37.2 ± 5.1	18	58	29	26.8 ± 1.4	39	61	60
**DIN flux**	1.2 ± 0.1	53	342	160	1.3 ± 0.1	176	122	72

Values of flux rates are given as average ± SE (n = 3).

### Initial colonization of benthic fauna in the Gyldensteen coastal lagoon

**Fig 5:**
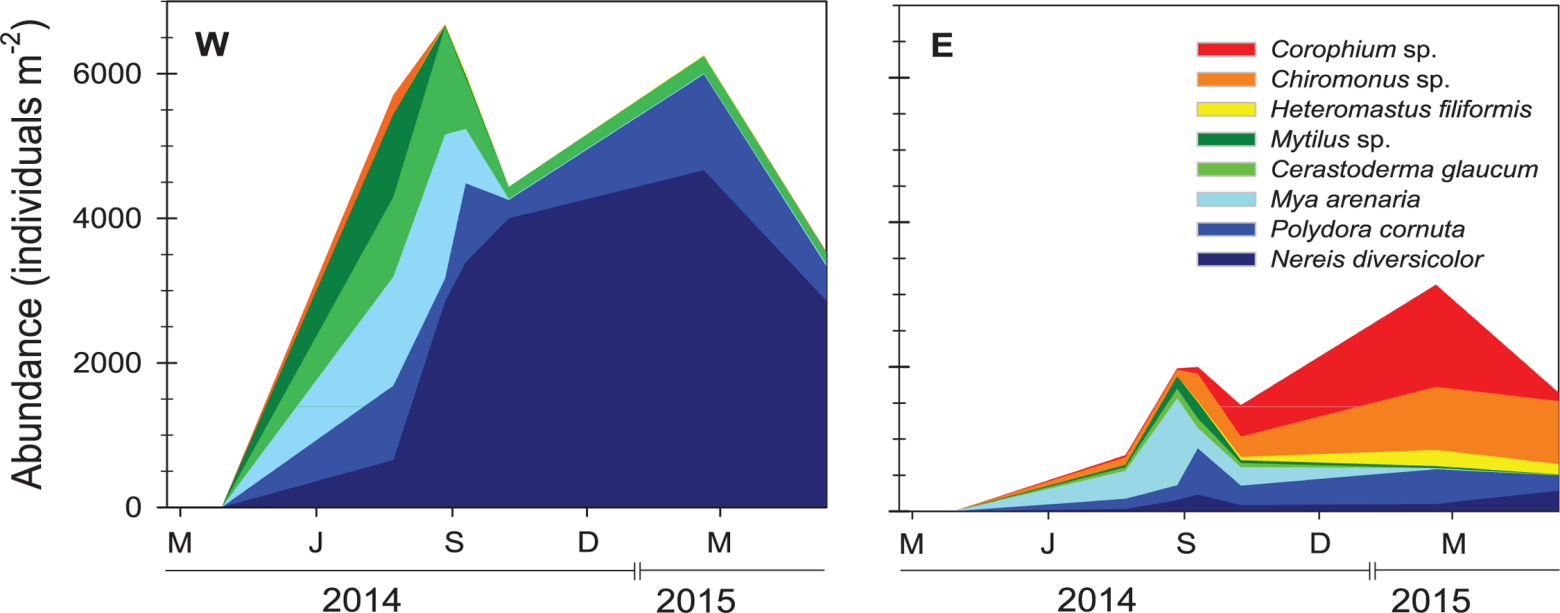
Colonization of benthic infauna at the Western (W) and Eastern (E) stations after flooding of the Gyldensteen Coastal Lagoon. March 29, 2014 is point of origin for all graphs. X-axis tick marks indicate start of March (M), June (J), September (S) and December (D).

Of the 12 pioneering benthic macrofaunal species entering the lagoon in 2014 and 2015, 8 species were at times considered dominant (Fig 5). Colonization occurred rapidly during the first year in the newly flooded lagoon with shifting dominance between different groups of marine benthic fauna. Stations W and E showed similar colonization patterns, but colonization was delayed and abundances were generally lower at station E (Fig 5). Total fauna abundance at station W varied from 3600 to 6800 ind m^-2^ while station E ranged from 1000 to 2300 ind m^-2^. Bivalves (i.e. *Cerastoderma glaucum*, *Mya arenaria* and *Mytilus edulis*) were the first dominating group that occupied the lagoon at abundances of ~1500 (E) and ~3800 (W) ind m^-2^ (65–76% of total benthic fauna) 4–5 months after the flooding. However, the bivalve community crashed in September 2014, and subsequently their abundance never exceeded 350 ind m^-2^ in the study period. Simultaneously, polychaetes proliferated and attained relatively stable abundances of ~500 (E) and ~4000 (W) ind m^-2^ towards the end. Only two species of polychaetes, *Nereis diversicolor* and *Polydora cornuta*, were consistently found in high numbers, while species like *Nereis (Alitta) virens*, *Nereis (Alitta) succinea*, *Pygospio elegans*, and *Capitella capitata* were less abundant. Few species were more abundant in station E than station W, including the polychaete *Heteromastus filiformis*, the insect larvae *Chironomus* sp. and the crustacean *Corophium* sp. The remaining species observed at both stations were only present in low numbers and consisted of gastropods (*Hydrobia* sp. and *Littorina littorea*). The fauna abundance in UC soil (890 ± 419 ind m^-2^) was comparable with station E, but lower than station W. The fauna at UC was dominated by *N*. *diversicolor* (84%), followed by *N*. *succinea* (8%) and *N*. *virens* (5%).

The benthic fauna communities outside the lagoon, stations OW and OE, consisted of 13 species and were dominated by polychaetes (~2600 ind m^-2^, 96% of total fauna). They were mostly represented by *N*. *diversicolor*, *M*. *viridis*, *C*. *capitata* and *H*. *filiformis* with individual abundances ranging from average 180 to 920 ind m^-2^. Other common species such as *S*. *armiger* and *A*. *marina* were found in abundances up to 19 and 28 ind m^-2^, respectively. Other invertebrate groups such as oligochaetes, bivalves, gastropods and crustaceans only accounted for 1–2% of total fauna.

**Fig 6:**
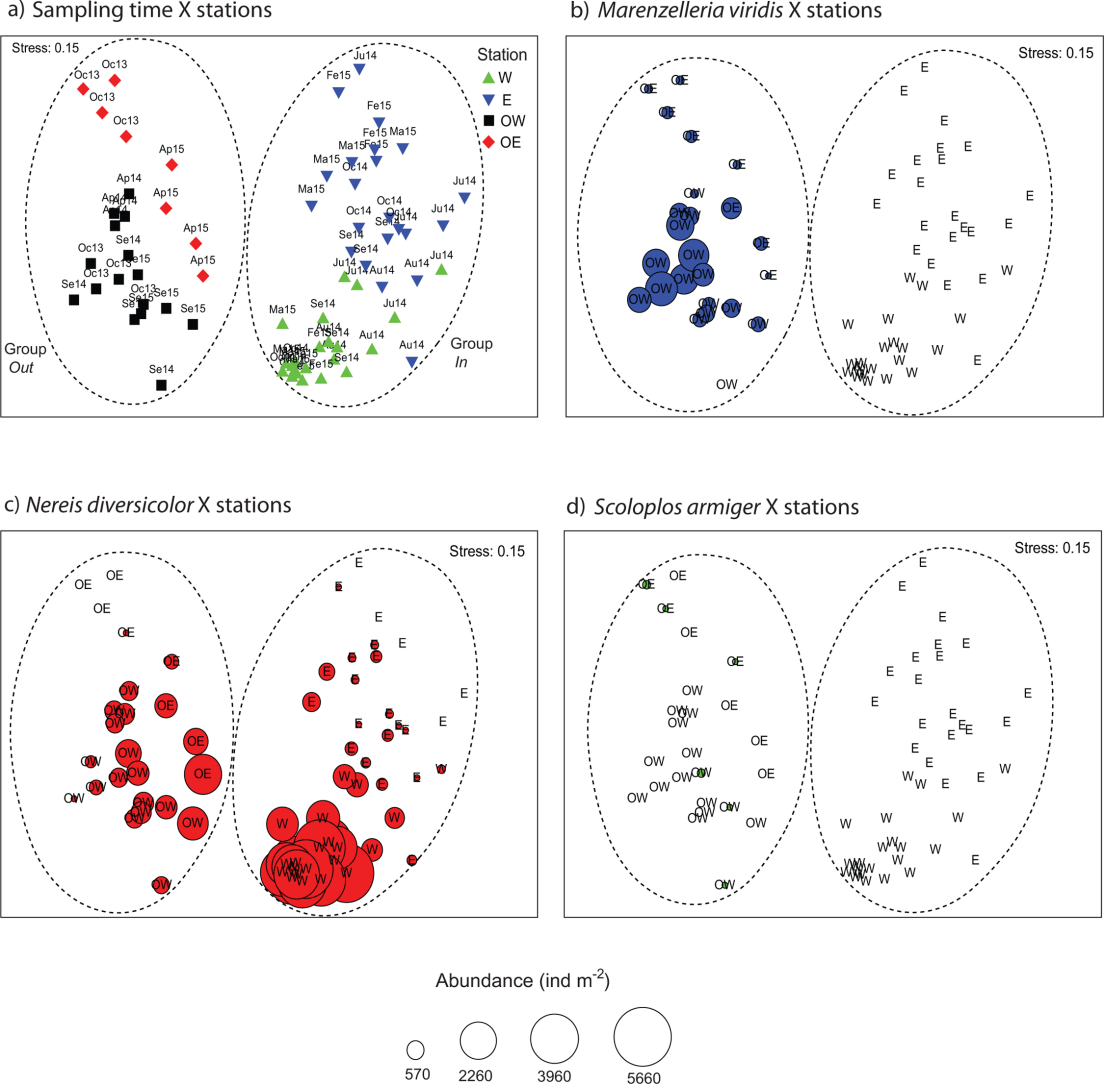
Two-dimensional plots of MDS outputs based on Bray-Curtis similarity matrix. Panel (a): distribution of fauna dataset indicated by time and station. Time is represented as month by the two first letters and year by the two last numbers. Sampling months: February, April, May, July, August, September, and October. Sampling years: 2013 to 2015. Sampling stations: Western site inside the lagoon (W), Eastern site inside the lagoon (E), Western site outside the lagoon (OW), and Eastern site outside the lagoon (OE). The dashed ellipses indicate the groups of stations tested by ANOSIM analysis: Group *Out* composed of stations located outside the lagoon and Group *In* composed of stations inside the lagoon. Panels (b), (c) and (d): spatial and temporal abundance of species tested in the laboratory experiment.

There were no marked differences in macrofauna abundance and composition between sampling times for all stations according to MDS multivariate analysis (Fig 6). Instead, a clear grouping was observed of stations outside (Group *Out*: OW and OE) and inside (Group *In*: W and E) the lagoon (Fig 6). Within Group *In* there was separation between stations W and E (except in July and August 2014), while a corresponding separation between stations OW and OE within Group *Out* was not clearly observed due to a lower number of sampling times. The species composition of macrofauna between Group *In* and Group *Out* was significantly different as indicated by ANOSIM (Global R = 0.805, p = 0.001). Accordingly, SIMPER analysis indicated that Group *Out* and Group *In* had total average dissimilarity of 82%. The species that mostly contributed to this dissimilarity (~50% of the total) were *N*. *diversicolor*, *M*. *viridis*, *C*. *capitata* and *P*. *cornuta*. Group *Out* was dominated by the species *N*. *diversicolor*, *M*. *viridis*, *C*. *capitata* and *H*. *filiformis* which contributed 91% of the cumulative similarity within the group (Table 4). In Group *In*, *N*. *diversicolor* was also important and together with *P*. *cornuta*, *C*. *glaucum*, *Chironomus* sp. and *M*. *arenaria* also summed up to 91% cumulative similarity (Table 4). Furthermore, *N*. *diversicolor* was the only species selected for the laboratory experiment that succeeded in colonizing the lagoon within the study period (Fig 6). Despite the presence of *M*. *viridis* and *S*. *armiger* outside the lagoon, these species were not among the early colonizers (Fig 6).

**Table 4:** Abundance, average similarity (Av. Sim.) and contribution (Cont.) of the most important species to the similarity within Group *Out* and Group *In* based on SIMPER analysis.

	Group *Out*				Group *In*		
Species	Abundance (ind m^-2^)	Av. Sim. %	Cont. %	Species	Abundance (ind m^-2^)	Av. Sim. %	Cont. %
*Nereis diversicolor*	651 ± 116	16	32	*Nereis diversicolor*	1603 ± 280	14	31
*Marenzelleria viridis*	582 ± 106	15	32	*Polydora cornuta*	540 ± 88	11	25
*Capitella capitata*	924 ± 205	10	21	*Cerastoderma glaucum*	331 ± 80	6	12
*Heteromastus filiformis*	179 ± 62	3	6	*Chironomus* sp.	356 ± 69	5	12
				*Mya arenaria*	506 ± 105	5	11
**Total cumulative %**		44	91			41	91

Abundance values represent mean ± standard error.

## Discussion

### Polychaete behavior in flooded soil

Danish coastal marine sediments are typically homogenous to 10–20 cm depth and usually consist of well-sorted fine-to-medium sands, with varying content of silt-clay particles and medium porosities of 0.3–0.6 [40]. These characteristics fit well with those below the vegetated surface of the flooded C soil at Gyldensteen Coastal Lagoon (Table 1), suggesting that physical manipulation through ploughing throughout the land use period had not modified the soil beyond recognition. The physical mixing and aeration also assured efficient organic matter degradation, as organic carbon levels were relatively low and comparable with other marine coastal areas [40]. However, the UC soil had over the same time frame developed a strong terrestrial structure, with a surface layer containing dense networks of plant roots and a dense clay layer below.

Overall, the tested polychaete species showed good short-term survival and were capable of forming burrows in both types of flooded soil, as no significant difference in recoveries was detected between soil treatments. The 28–89% recovery found for all the species is within the range typically found for similar laboratory experiments that add fauna to coastal sediments [17,41]. Thus, none of the three species could be eliminated as potential colonizers of the newly flooded lagoon. However, out of the three tested species, *M*. *viridis* was apparently the least likely colonizer as it showed a lower recovery (28–44%) and higher 42% avoidance behavior (i.e. escaping number of individuals) when exposed to flooded soils. The high survival of *N*. *diversicolor* in flooded soils corresponded well with its status as a pioneer species, capable of inhabiting stressful habitats in estuaries and along the open coast [42,43]. Likewise, the high survival of *S*. *armiger* in flooded soils is in agreement with its cosmopolitan distribution and ability to dwell in contrasting substrata [34].

The measurements of bioirrigation intensity and inconsistent shape of porewater SO_4_^2-^ and Br^-^ profiles indicate that the two soil types may have led to diverging behavioral responses of the fauna. The deeper penetration and higher SO_4_^2-^ and Br^-^ concentrations in faunated C than in UC soil shows faster porewater transport via well-functioning burrows in the former substrate. Accordingly, the significantly higher area-specific bioirrigation at rates similar to those reported from marine sediments [30,44] in C compared to UC soil, suggests that soil texture and composition influenced the performance of the species. The lower organic content and the sandy structure of the C soil, which is typical for coastal marine sediments, seemed to favor the natural ecological behavior of the three tested species. In contrast, the compact clay layer at 5–10 cm depth in UC soil was clearly a physical barrier towards deep bioirrigation as indicated by the narrow porewater SO_4_^2-^ and Br^-^ profiles. Furthermore, the unconsolidated structure of the upper peat-like root-layer in UC soil with high 70–80% water content and porosity of 0.8 probably posed an additional obstacle for construction of optimal burrows decreasing the hydraulic resistance for efficient burrow ventilation [45].

### Impacts of fauna on biogeochemical cycling in flooded soil

The flooding of cores in the experimental set-up undoubtedly led to a dramatic shift in soil biogeochemistry, including a shift from oxidized to reduced conditions [33] since anaerobic sulfate reduction dominate organic matter degradation in seawater saturated substrata [27]. Consequently, flooding annihilated obligate aerobic soil organisms, while microorganisms tolerant to anoxic and saline conditions prospered [27]. The relatively high metabolism in the defaunated soil treatment about one month after flooding and onward suggests rapid organic matter degradation by the newly developed heterotrophic microbial community. This is in accordance with previous experiments showing that heterotrophic microbial communities in soils and sediments respond within weeks to flooding with seawater and changes in porewater SO_4_^2-^ concentrations [27,46–48]. Interestingly, the levels of SOU and fluxes of TCO_2_ in faunated soils were comparable to *in situ* rates observed during spring (10–12°C) in nearby estuaries such as Odense Fjord, while DIN efflux was 2–4 fold increased [7]. Species-specific effects on solute exchange were more evident for SOU, where *N*. *diversicolor* and *S*. *armiger* both tended to stimulate microbial activity more than did *M*. *viridis*. These results can be explained by the performance of the species, since *N*. *diversicolor* and *S*. *armiger* were more successful colonizers than *M*. *viridis* in both soil types. However, due to specific characteristics of the soils, the different behavior of the worms did not always result in sharp species-specific responses of the other solute fluxes.

The generally higher TCO_2_ fluxes and SOU in UC than C soil were likely caused by higher content of labile organic matter in the former. More importantly, rates in all treatments with polychaetes were stimulated (18–105%) compared with defaunated controls (Fig 4 and Table 3), suggesting that the infaunal activity stimulated organic matter degradation regardless of soil type. An ‘infaunal effect’ on benthic metabolism of the same magnitude has frequently been observed in marine sediments [17,49,50]. Most (70–90%) of the stimulation is usually due to enhanced microbial rates, while respiration by the fauna accounts for the rest [18,51].

**Fig 7:**
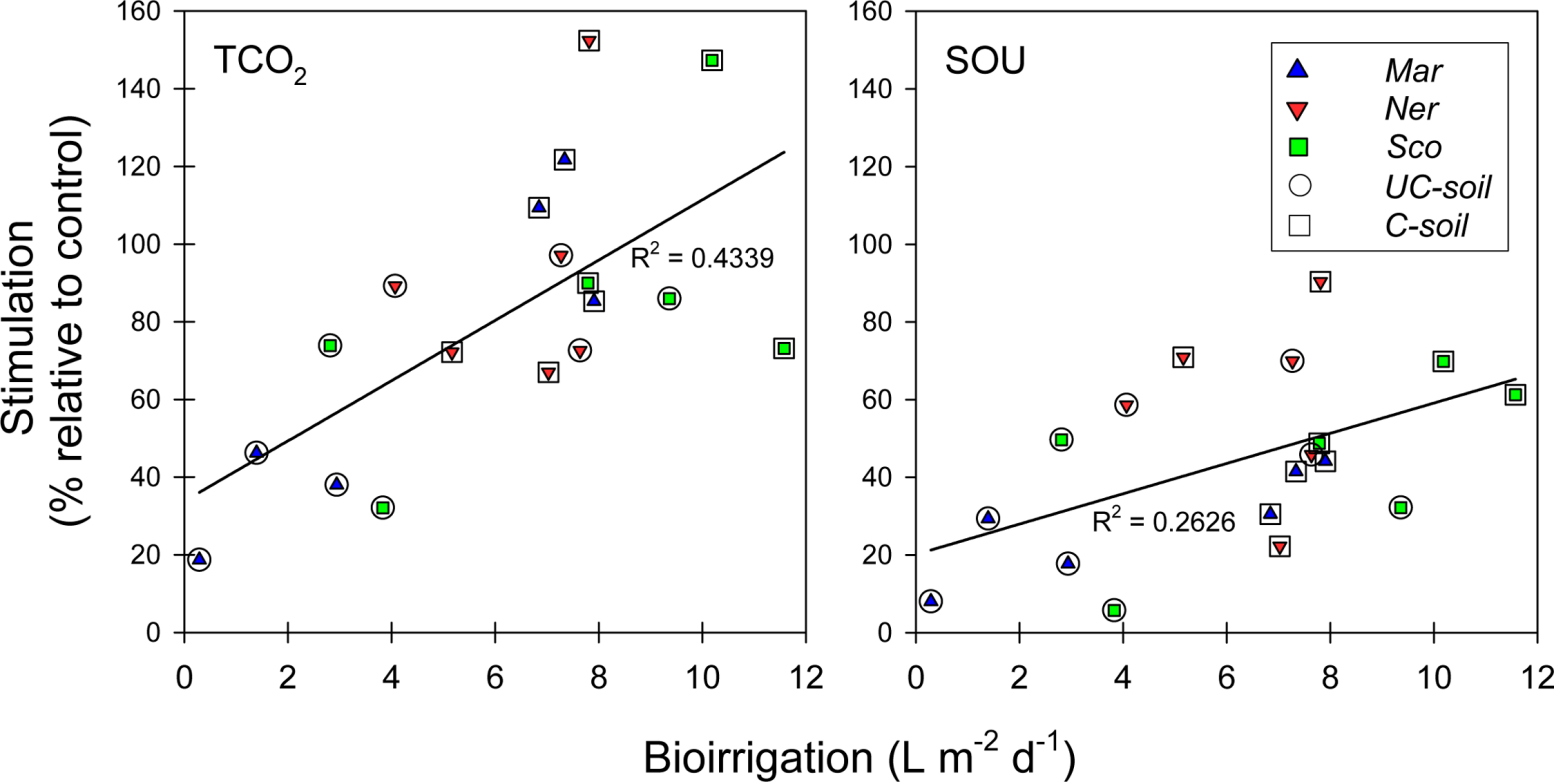
Linear regressions between the relative stimulation of TCO_2_ efflux and sediment O_2_ uptake (SOU) and bioirrigation intensity in flooded uncultivated (UC) and cultivated (C) soils with polychaetes added (Mar: *Marenzelleria viridis*, Ner: *Nereis diversicolor* and Sco: *Scoloplos armiger*).

Stimulated microbial processes are a consequence of complex interactions coupled to burrow structures and ventilation behavior of the resident infauna [11]. Increased O_2_ availability at depths where anoxic conditions otherwise prevail stimulates the degradation of refractory organic matter, which otherwise is non-degradable under anoxic conditions [16,52]. Similarly, ventilation driven solute transport affects the distribution of essential electron acceptors [53,54], and the same effect was evident from the elevated porewater SO_4_^2-^ concentrations in soils with polychaetes in this experiment. Burrow-dwelling polychaetes thus ensure that microbial degradation can proceed at high rates and under non-limiting conditions in the entire bioturbated zone. This is substantiated by the significant positive relationships between bioirrigation intensity and TCO_2_ effluxes and SOU, suggesting that the presence of infauna exerts a critical control on sediment metabolism and solute exchange through the overlying water (p = 0.003 and 0.029, respectively; Fig 7).

Microbial degradation of organic N and P usually occurs in proportion to degradation of organic C, reflecting the stoichiometric C:N:P ratios in the organic matter being degraded [46,55]. Fauna stimulation of DIN fluxes in the newly flooded soils followed the overall trends described for TCO_2_ fluxes (Fig 4 and Table 3). However, the enhancement of DIN fluxes exceeded in most cases that observed for TCO_2_ fluxes. Thus, *N*. *diversicolor* stimulation of DIN efflux in UC soil (342%) was four times higher than observed for TCO_2_ fluxes. The excess of DIN fluxes is probably a consequence of substantial NH_4_^+^ desorption and changes in nitrification-denitrification coupling [56] due to ventilation-driven porewater flushing. No DIP efflux was detected in any of the treatments, suggesting high P-retention in the flooded soils. In fact, both soil types accumulated extremely high near-surface Fe(III) levels of ~60–160 µM cm^-3^ during the first year after flooding; one order of magnitude higher than levels normally found in Danish coastal areas [27,57]. This Fe(III)-rich P-buffer in the soils is generated by oxidation of dissolved Fe^2+^ diffusing towards the soil surface from the newly anoxic deeper layers where soil Fe(III) has been reduced [58].

This study suggests that early colonizing polychaetes have significant stimulatory effects on structure and biogeochemical processes in newly flooded soils. They may be crucial for the development of essential ecological functions, since burrows and active bioturbation increases the connectivity between the soils and the overlying water column. Burrow ventilation activities stimulate solute transport and enhance organic matter degradation, constituting a critical control on soil chemical conditions, water column nutrient levels and overall productivity of newly flooded ecosystems.

### Benthic fauna colonization in the Gyldensteen coastal lagoon after flooding

The W and E stations sampled for fauna colonization in Gyldensteen Coastal Lagoon had soil characteristics similar to the C soil from the laboratory experiment, where the three tested polychaete species performed well. Nevertheless, only *N*. *diversicolor* successfully colonized the lagoon within the first year. This species even managed to colonize UC soil in abundances (749 ± 338 ind m^-2^) comparable to E. However, due to the lack of a temporal analysis, we cannot confirm the colonization pattern of *N*. *diversicolor* and other benthic species in UC soil. The early colonizers of *N*. *diversicolor* in the flooded agricultural soils probably arrived as juveniles and adults via bedload transport from the adjacent marine environments [23,32,59], while *M*. *viridis* and *S*. *armiger* were for unknown reasons not among the early colonizers. The avoidance behavior of *M*. *viridis* adults, i.e. by swimming away from flooded soils in the experimental set-up, may indicate that this species was deterred from entering the lagoon. Swimming ability of *Marenzelleria* species has been observed before and is associated to worms repositioning themselves with regard to incompatible environmental conditions [60]. Furthermore, *M*. *viridis* and *S*. *armiger* are known to live in close association to *A*. *marina*, which was not found as colonizer inside the lagoon. *A*. *marina* is known as an ecosystem engineer and is a typical member of the community outside the lagoon. It intensively reworks the sediment forming well-sorted and permeable sand by burying coarse particles > 1 mm below 10–20 cm depth and removing silt+clay by resuspension [61]. These mechanisms, together with downward oxygen transport through vigorous ventilation, make conditions suitable for *M*. *viridis* and *S*. *armiger* [43,62]. On the other hand, the laboratory experiment tested the performance of adult individuals, while the colonizing stages of *M*. *viridis* and *S*. *armiger* polychaetes are predominantly benthic or pelagic larvae [28,34,63]. Therefore, the reason why these species were not among the early colonizers in Gyldensteen Coastal Lagoon could also be low tolerance of larvae and juveniles to newly flooded soils.

The flooded agricultural soils in Gyldensteen Coastal Lagoon did not impose a challenging substratum for several other macrofaunal colonizers. The invasion by these occurred rapidly in the first year and they reached similar or higher levels than outside the lagoon. This is probably a consequence of empty niches, excess resources and lack of competition, which cause rapid population expansion of opportunistic species [64]. The initial dominance of juvenile *C*. *glaucum* and *M*. *arenaria* is one example, as larvae of these bivalves settled almost immediately after the flooding in spring and proliferated in an environment without potential predators and with plenty of food. The settling corresponded exactly with the time when these species have pelagic larvae [65–67]. Although abundance was higher at station W, early colonization patterns at W and E stations were similar in terms of species composition. The lower abundance and slower colonization at E was partly caused by longer distance from the main entrance in the west and partly by anoxia, which was consequence of a massive bloom of green macroalgae observed as a single event in E during summer 2014 [68].

The polychaetes *N*. *diversicolor*, *M*. *viridis* and *C*. *capitata* were dominant and among the species that contributed most to the structural difference outside compared with inside the lagoon (Table 4). While *N*. *diversicolor* had 2-fold higher abundance inside than outside the lagoon, *C*. *capitata* was found in low abundances and *M*. *viridis* completely absent inside the lagoon. Successful establishment of *M*. *viridis* and *C*. *capitata* is thus critical for a benthic community with a structure and functioning comparable to ambient marine communities. The dominance of *N*. *diversicolor* inside the lagoon corroborates with other findings of *N*. *diversicolor* as one of the first species of benthic infauna to colonize newly formed salt marshes and tidal flats [9,22]. Although *C*. *capitata* and other capitellid species such as *H*. *filiformis* were present in low abundances inside the lagoon, they should have the potential to develop into higher abundances, as normally found for these rapid colonizers during early succession of disturbed areas [69]. The small tube building polychaete *Polydora cornuta* is another typical opportunistic species [70] that appeared as a dominant species during the early succession in the lagoon (Table 4). In fact, *P*. *cornuta* and sibling species have often been identified as early colonizers in restored salt marshes and mudflats [4,9,22,23].

The polychaete *N*. *diversicolor* with U-shaped burrows and vertical tube builders such as the capitellids and *Polydora* are well-known to extend the depth of oxidized sediment and stimulate benthic metabolism, which improves environmental conditions for the recruitment of other species [51,70,71]. The laboratory experiment showed that the successful early colonizers *N*. *diversicolor* and *S*. *armiger* (with I-shaped vertical burrow) increased carbon mineralization (i.e. TCO_2_ release) by 97–103% in C soils. Considering, for instance, the species-specific effects of *N*. *diversicolor* on TCO_2_ release (i.e. TCO_2_ release per individual = 80 µmol m^-2^ d^-1^) and its average abundance in the lagoon (1603 ind m^-2^), we estimate that organic carbon degradation was increased by 219% in the first year of flooding. Furthermore, burrow ventilation by the dominant *N*. *diversicolor* stimulates the degradation of aged, refractory organic matter [71] and may influence the pools of organic matter buried in anoxic subsurface soils. These examples show that bioturbation of pioneering benthic fauna may play an important role by modifying the chemical and organic conditions in soils, which is critical for the continued benthic community succession in the newly flooded lagoon.

## Conclusions

Several important conclusions regarding biological and ecological developments in newly flooded coastal ecosystems can be made based on this study. Laboratory experiments showed that adult polychaetes (three species tested: *Marenzelleria viridis*, *Nereis diversicolor* and *Scoloplos armiger*) can readily colonize, survive and perform vital functions (ventilation and bioirrigation) in terrestrial soils flooded with seawater. This conclusion was partly verified by *in situ* observations in the newly flooded Gyldensteen Coastal Lagoon, where rapid colonization by *N*. *diversicolor* and other benthic fauna species, including *Polydora cornuta*, was observed, while *M*. *viridis* and *S*. *armiger* were not yet present. The early colonizers probably modify the soil conditions before further benthic community succession, including *M*. *viridis* and *S*. *armiger*, will be evident inside the lagoon. We therefore conclude that early colonizing polychaetes exert a critical control on ecological developments in newly flooded coastal ecosystems, since they provide the essential improvement of environmental conditions required for the recruitment of other species. We expect that the functional effects of species succession, e.g. stimulation of C cycling, will lead to increases in biodiversity and more complex food-webs with the appearance of larger invertebrates (i.e. shellfish, crabs), fishes and birds [4,12,72]. As opposed to other managed flooded wetlands, e.g. saltmarshes [4,72], a large part of Gyldensteen Coastal Lagoon soil has similar water depth and its soil composition is relatively homogeneous due to its previously marine origin and many years of agricultural activity. This is probably an extra advantage in terms of colonization and establishment of benthic fauna. Low-lying land and reclaimed agricultural soils protected by dikes may thus have strong potential to become well-functioning marine coastal lagoons with high ecological and recreational value.
